# Sex and Sleep Quality Effects on the Relationship Between Sleep Disruption and Pain Sensitivity

**DOI:** 10.1002/ejp.70023

**Published:** 2025-04-08

**Authors:** Elisabet Dortea Ragnvaldsdóttir Joensen, Laura Frederiksen, Signe Vindbæk Frederiksen, Emilie Stjernholm Valeur, Rocco Giordano, Emma Hertel, Kristian Kjær‐Staal Petersen

**Affiliations:** ^1^ Faculty of Medicine, Aalborg University Aalborg Denmark; ^2^ Department of Health Science and Technology, Faculty of Medicine, Center for Neuroplasticity and Pain (CNAP), Aalborg University Aalborg Denmark; ^3^ Department of Oral and Maxillofacial Surgery Aalborg University Hospital Aalborg Denmark; ^4^ Department of Materials and Production, Center for Mathematical Modeling of Knee Osteoarthritis (MathKOA) Aalborg University Aalborg Denmark

## Abstract

**Background:**

Chronic pain affects around 20% of the global population and is influenced by various factors, including sleep quality. Studies indicate that sleep disruption can enhance pain sensitivity; however, it is unclear how sex and baseline sleep quality impact these findings. This study examines how sex and baseline sleep quality impact the effects of three nights of sleep disruption on pain sensitivity in healthy individuals.

**Methods:**

Fifty‐nine participants (30 females) underwent two laboratory sessions, separated by three nights of sleep disruption. Pain sensitivity was measured using cuff and handheld algometry, and participants completed a battery of questionnaires on sleep quality, positive and negative affect, and pain catastrophising. Sleep patterns were collected through wrist actigraphy and self‐reported sleep diaries.

**Results:**

Temporal summation of pain was significantly facilitated in males (*p* < 0.01), and pain during suprathreshold stimulation was increased for females (*p* < 0.01) after the experimental sleep disruption. No differences in any QST parameters were found when comparing participants with good or poor sleep at baseline, but those with good baseline sleep rated the suprathreshold stimulation as more painful (*p* < 0.05) after the experimental sleep disruption. Finally, having good or poor sleep quality at baseline was associated with a significant reduction in self‐reported sleep quality and level of rest after the experimental sleep disruption (*p* < 0.05).

**Conclusion:**

This study indicates that sleep disruption might impact sexes differently and indicates that prior sleep quality is less likely to impact this.

**Significance:**

Sleep disruption protocols can mimic the sleep problems experienced by patients with chronic pain. The current study explains how different sexes respond to a 3‐night sleep disruption protocol and explains how sleep quality at baseline might impact these results.

## 1 Introduction

Chronic pain affects approximately 20% of the global population (Hadi et al. 2019) and is characterised by significant individual variability in pain intensity (Pagé et al. 2022). Poor sleep quality is commonly observed in patients with chronic pain (Hadi et al. 2019; Tang 2008), and studies suggest that poor sleep quality increases clinical pain intensity (Boye Larsen et al. 2021; Finan et al. 2013). Experimental sleep disturbance protocols may increase experimental pain intensity in healthy individuals (Chang et al. 2022). Previous studies have aimed to address this relationship using experimentally induced sleep disturbances (Hertel et al. 2023; Iacovides et al. 2017; Staffe et al. 2019). These experimentally induced sleep disturbances have been found to impact central pain mechanisms (Hertel et al. 2023; Smith et al. 2007; Staffe et al. 2019) and psychological parameters (Hertel et al. 2023; Stenson et al. 2021), highlighting the link between poor sleep quality and pain. A large population study found that 57.9% of young adults have poor sleep quality (Fatima et al. 2016), but this is rarely considered during recruitment and data analysis in experimental sleep studies. Individuals already presenting with poor sleep quality might respond differently to experimental sleep disruptions, but this has not been investigated in previous research. Furthermore, females are twice as likely to report poor sleep quality compared to males (Madrid‐Valero et al. 2017). A recent systematic review and meta‐analysis found that females demonstrated increased pain sensitivity compared to males following sleep provocation protocols (Rouhi et al. 2023), but the analysis was based on four smaller studies and limited by lack of sex‐based separation in the pain sensitivity measures, and therefore more evidence is needed to confirm this.

Quantitative sensory testing (QST) is used in the assessment of sensory function for studying musculoskeletal pain, pressure pain threshold (PPT) and pain tolerance threshold (PTT), as well as dynamic measures like temporal summation of pain (TSP), a proxy assessment of spinal excitability (Arendt‐Nielsen and Graven‐Nielsen 2011), and conditioned pain modulation (CPM), a proxy measure of the descending inhibitory control system (Cummins et al. 2020) Studies suggest that patients with chronic musculoskeletal pain exhibit facilitated TSP and impaired CPM compared to healthy subjects (Arendt‐Nielsen et al. 2015; McPhee et al. 2020). Moreover, TSP and CPM measures have high between‐person variability, and this variability has been linked to treatment outcomes (Petersen et al. 2021), and therefore it is important to understand some of the factors that impact this variability.

Previous studies examining the effects of sleep disturbance protocols on QST in healthy individuals have shown significant heterogeneity (Chang et al. 2022; Herrero Babiloni et al. 2024), possibly due to inter‐individual variability in included participants, such as differences in sex and baseline sleep quality. Further, most previous experimental sleep protocols either apply exploratory total sleep deprivation or sleep restrictions, which might not mimic the sleep pattern experienced by patients with chronic pain, where the most common change is disruptions of sleep continuity (Bjurstrom and Irwin 2016). Therefore, the current exploratory study aimed to assess how sex and baseline sleep quality impact QST outcomes before and after three nights of sleep continuity disruption in healthy participants. We hypothesised that being female and having poor sleep quality at baseline would increase the effect of experimental sleep disruption.

## 2 Methods

**FIGURE 1:**
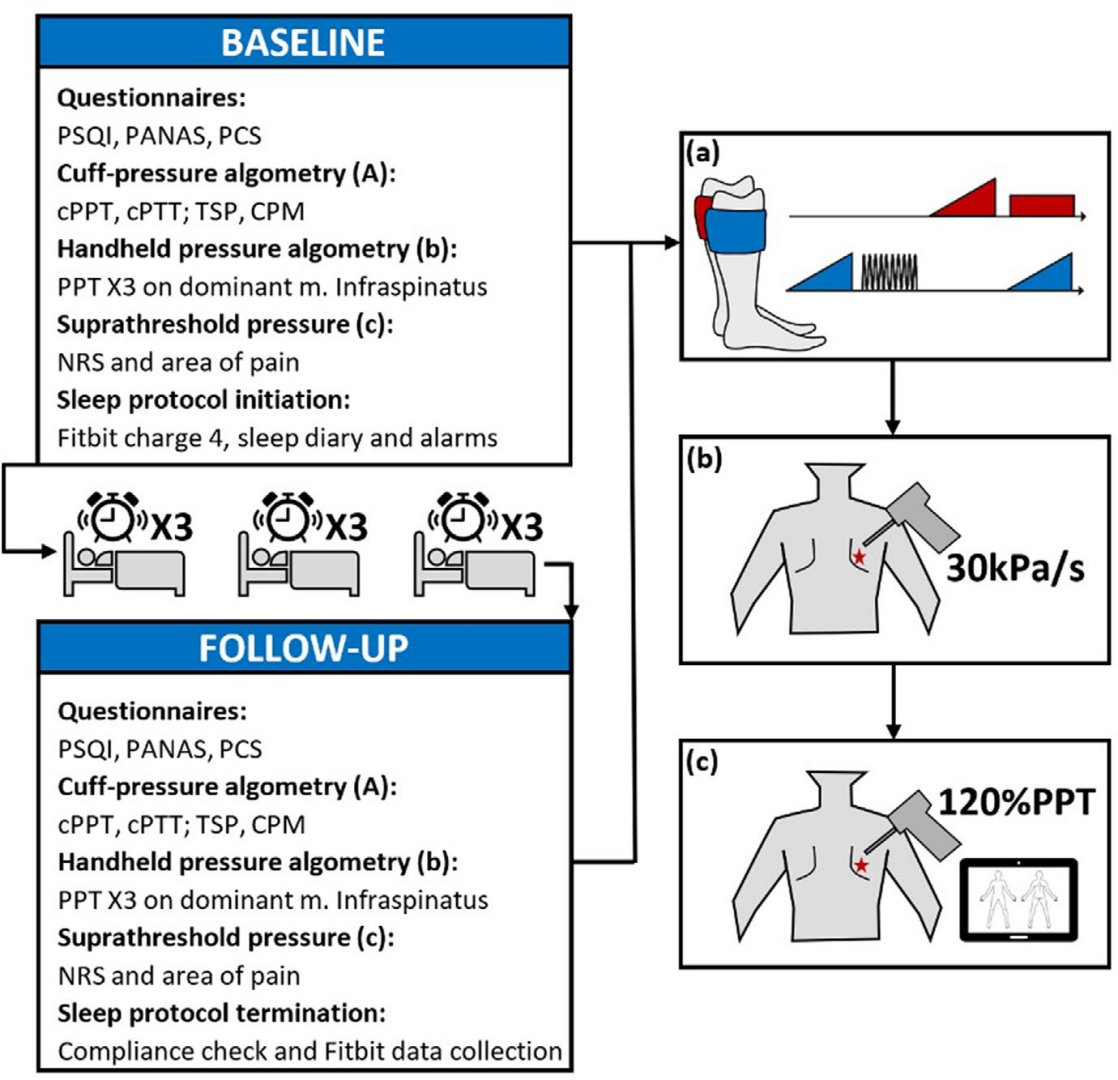
Overview of the experimental setup, illustrating a timeline of baseline, sleep disruption, and follow‐up. (A) Illustration of cuff algometry with measurements of cuff pressure pain thresholds (cPPT) and cuff PTT (cPTT), measured by ramped inflations of 1 kPa/s; temporal summation of pain (TSP), measured with 10 consecutive inflations at the pressure of PTT on the dominant leg with a rate of 100 kPa/s with 1 s intervals between stimuli; and conditioned pain modulation (CPM), measured by the application of a constant stimulus at 70% PTT and a ramped inflation of 1 kPa/s. (B) Handheld algometry pressure, applied three times on the dominant musculus infraspinatus. (C) 120% suprathreshold stimulus, applied to the dominant infraspinatus muscle, first for 5 s and then for 60s. NRS, Numeric Rating Scale; PANAS, The Positive and Negative Affective Schedule; PCS, Pain Catastrophizing Scale; PSQI, Pittsburgh Sleep Quality Index.

This exploratory study included two laboratory sessions separated by three nights of sleep disruption. Both sessions were conducted at Aalborg University, Aalborg, Denmark, and lasted approximately 1.5 h. During each session, participants completed a series of validated questionnaires and underwent pain sensitivity assessments using computer‐controlled cuff‐pressure algometry and handheld algometry. During the baseline session, participants received a Fitbit Charge 4 to wear on their non‐dominant wrist for the entire study period. Participants were informed about the planned awakenings and instructed to set alarms for the scheduled wake‐up times. Participants completed a sleep diary at baseline and on every morning following the three consecutive nights of sleep disruption. Figure 1 provides an overview of the experimental protocol.

### 2.1 Participants

Fifty‐nine healthy ethnic Danes of Caucasian ancestry (30 females) aged 18–45 years were recruited. This was done through Aalborg University, Forsog.dk, and social media. The participants were excluded if they reported one of the following: pregnancy, drug addiction, previous or current neurological or musculoskeletal illnesses, current pain, or lack of ability to cooperate. Qualified participants were provided with written and verbal information regarding the study and signed an informed consent prior to enrollment. Four participants were excluded due to lack of ability to cooperate by not completing the sleep disruption protocol. This study was approved by the local Ethics Committee (N‐20230062 and N‐20180089) and conducted in accordance with the Helsinki Declaration.

### 2.2 QST

#### 2.2.1 Computer‐Controlled Cuff‐Pressure Algometry

A computer‐controlled cuff algometer (Cortex Technology, Denmark) was used to investigate deep‐tissue pain sensitivity in a standardised manner. This method has good‐to‐excellent reliability, which is comparable to or better than other available QST tools (Graven‐Nielsen et al. 2017; Imai et al. 2016; Petersen et al. 2017; Vaegter et al. 2018). The machine was equipped with two 13 cm wide air‐filled tourniquet cuffs (VBM Medical) positioned at the widest part of each calf. An electronic visual analog scale (eVAS) measuring 10 cm, ranging from 0, no pain, to 10, the worst pain imaginable, was used to estimate pain intensities.

##### 2.2.1.1 Cuff PPT (cPPT) and Tolerance Threshold

cPPT and cuff tolerance threshold (cPTT) were investigated on the dominant leg. The participants were instructed to continuously rate the intensity of the pain on the eVAS and to press the stop button when the tolerance threshold was reached, immediately releasing the pressure. The measurements were conducted with a 1 kPa/s pressure increase with a 100 kPa safety cut‐off. The cPPT was defined as the point where the stimulus became painful, indicated by the eVAS score exceeding 1 cm, and the cPTT was defined as the pressure where the pain was no longer tolerated, leading the participants to press the stop button. These definitions were used based on previous studies using similar methods (Hertel et al. 2024; Petersen et al. 2016, 2019).

##### 2.2.1.2 Temporal Summation of Pain

Temporal summation of pain was assessed by applying 10 repeated inflations on the dominant leg at the pressure level determined as the cPTT. Each inflation lasted 1 s and was separated by one‐second inter‐stimulus intervals. The participants were instructed to continuously rate the pain intensity on the eVAS for each inflation, without returning to zero between the stimuli. The pain intensity rated on the eVAS was noted for each of the stimuli, and TSP was calculated as the difference between the average score of the last three eVAS scores and the average of the first four eVAS scores (the average of the last three subtracted from the average of the first four), based on previous literature (Petersen et al. 2020).

##### 2.2.1.3 CPM

To assess CPM, a conditioning stimulus was applied to the non‐dominant leg, corresponding to 70% of the pressure for cPTT on the non‐dominant leg, while a test stimulus was applied to the dominant leg, which gradually increased by 1 kPa/s with a 100 kPa safety cut‐off. The participants were instructed to continuously rate the intensity of the pain and to press the stop button when their tolerance was reached. CPM was then calculated as the difference between the cPPT during the conditioning stimulus and cPPT without conditioning (cPPT during CPM subtracted from cPPT without conditioning). A positive CPM score indicated an efficient CPM, and a negative CPM score indicated an inefficient CPM (Hertel et al. 2023, 2024; Petersen et al. 2020).

#### 2.2.2 Handheld Pressure Algometry

A handheld pressure algometer (Somedic) was used to assess PPT on the dominant infraspinatus muscle. The algometer was equipped with a 1 cm^2^ rubber probe placed on the participants' infraspinatus muscle perpendicular to the body surface. The pressure was gradually increased by approximately 30 kPa/s. The participants were instructed to press a stop button when the pressure elicited a painful sensation, which was defined as the PPT. The measurements were repeated thrice, and an average was calculated. The average PPT was used to calculate 120% PPT for the subsequent suprathreshold pressure pain stimulus.

#### 2.2.3 Suprathreshold Pressure

To assess the participants' sensitivity to a suprathreshold pressure, a constant pressure of 120% PPT was applied to the dominant infraspinatus muscle. The suprathreshold pressure stimulation was performed twice, first for 5 s and then for 60 s. The pressure and duration of the suprathreshold pressure were chosen in accordance with previous literature (Bellosta‐López et al. 2023; Doménech‐García et al. 2022). Following each of the pressure stimulations, the participants were instructed to give a verbal pain intensity rating using a Numeric Rating Scale (NRS) (0, no pain at all, and 100, worst pain imaginable) and mark the area of the pain on a body chart. The mean ratio of marked pixels was calculated with the Pain Distribution Software developed by Kanellopoulus et al. (2021).

### 2.3 Questionnaires

A battery of validated self‐reporting questionnaires was used to assess the participants' sleep quality, positive and negative affect, and thoughts and feelings about pain. These were administered at baseline and after the sleep disruption. After the sleep disruption, an instruction was added to the questionnaires with long retrospective timeframes to ‘pay in mind the days and nights during the study’. The participants were also asked for demographic information including age, weight, and height.

#### 2.3.1 Pittsburgh Sleep Quality Index

The Pittsburgh Sleep Quality Index (PSQI) was used to assess the participants' sleep quality at baseline and follow‐up. PSQI consists of 19 items, generating seven component scores comprising: habitual sleep efficiency, sleep latency, sleep disturbances, sleep duration, use of sleep medication, subjective sleep quality, and daytime dysfunction during the past month. The seven component scores were scored on a 0–3 scale and all equally weighted. The seven scores were summed to a total PSQI score, thus ranging from 0 to 21, where a higher score implied a worse sleep quality (Buysse et al. 1989).

#### 2.3.2 Positive and Negative Affective Schedule

The Positive and Negative Affective Schedule (PANAS) was used to assess the participants' positive and negative affective states during the past week. PANAS is a tool used in experimental settings, which aims to assess the subjective well‐being of individuals by capturing their self‐reported experiences of positive and negative emotions (Roemer and Medvedev 2022). PANAS consists of 20 categories to describe the feelings and emotions of an individual, divided into two scales to determine the positive and negative affect separately. A lower score indicated a lower level, and higher scores indicated a higher level of either positive or negative affect (Díaz‐García et al. 2020).

#### 2.3.3 Pain Catastrophizing Scale

The pain catastrophizing scale (PCS) was used to assess the participant's catastrophizing of pain in general by considering different factors affecting the pain experienced by the individual. PCS considers three dimensions when assessing catastrophizing: rumination, magnification, and helplessness. A higher total score indicated a higher tendency of pain catastrophizing (Sullivan et al. 1995).

### 2.4 Wrist Actigraphy

The participants wore a wrist‐worn tracker (Fitbit Charge 4, Fitbit Inc.) on their non‐dominant wrist during the experiment. The Fitbit tracks daily activity and sleep. The data from the Fitbit was transferred to a mobile platform (Fitbit LLC) using Bluetooth. The primary use of the Fitbit in the current study was tracking sleep and awake states, and secondarily to assess sleep stages with minutes spent awake, in light sleep, deep sleep, and REM sleep. Fitbit Charge 4 is found to provide reasonable estimates of different sleep parameters such as sleep stages and awakenings (Schyvens et al. 2024).

### 2.5 Experimental Sleep Disruption

The protocol for the experimental sleep disruption included three consecutive nights of sleep disruption in the form of three awakenings to simulate the fragmented sleep patterns seen in patients with chronic pain (Bjurstrom and Irwin 2016).

The participants were instructed to set an alarm on their smartphone for each scheduled awakening (00:00, 02:30–03:00, and 05:00). Furthermore, the participants were instructed to send a photo of the Fitbit to the research team at each awakening to document being awake. If the emails were not sent, the Fitbit data served as a control to check compliance with the sleep protocol. Additionally, participants were asked about their compliance at the follow‐up session. Participants who slept through a maximum of two alarms during the intervention were still included in the study.

#### 2.5.1 Sleep Diary

Each morning, following the nights of sleep disruption, the participants were required to answer a short questionnaire concerning their sleep patterns during the previous night, e.g., when they went to bed, total time of sleep, and number of awakenings. Furthermore, the participants were asked to rate their sleep quality and level of rest, each on a scale from 0 to 100, where 0 indicated the worst quality imaginable and not rested at all, and 100 indicated the best quality imaginable and most rested possible. The participants were to fill out the sleep diary four times, once at baseline and after each of the three experimental nights.

### 2.6 Statistics

All data is presented as means (± standard deviation, SD) unless stated otherwise. Participants were grouped based on sex (male and female) and PSQI scores (good sleep: PSQI < 5 and poor sleep: PSQI ≥ 5) to investigate how the parameters affect the impact of the sleep disruption protocol. Independent sample t‐tests were used to compare baseline differences in the sex and PSQI groups. Repeated measures (RM) ANOVA was used to investigate the effect of sex and PSQI group on changes in QST measures, questionnaire scores, and sleep parameters over time. Sex and PSQI group were included as between‐subject factors in separate models and time as within‐subject factor in all cases. There were two levels for time in the comparison of QST measures and questionnaire scores at baseline and follow up; three levels of time in the comparison of the wrist actigraphy measures for the three experimental nights; and four levels of time in the comparison of sleep diary entries for baseline and the three experimental nights. Bonferroni‐corrected post hoc analyses were conducted when significant main effects were identified to clarify specific group differences. Assumptions were checked using appropriate statistical and visual methods. Assumptions of sphericity were tested using Mauchly's test of sphericity and the Greenhouse–Geisser correction was applied in cases of violation.

Statistical analyses were performed in SPSS statistics (IBM SPSS Statistics for Windows, Version 28.0). Significance was accepted at *p* < 0.05.

## 3 Results

### 3.1 Participant Characteristics

The exploratory current study included 59 participants (30 females) with a mean age of 24.4 (± 3.3) and a mean BMI of 24.0 (± 3.0). Ten participants slept through one alarm, and one participant slept through two alarms during the three nights of sleep disruption.

#### 3.1.1 Cohort Differences in Outcomes After Sleep Disruption

Paired‐sample t‐test found significantly decreased PTTs (*t*_58_ = 2.38, *p* < 0.05) and increased NRS rating during 120%PPT stimulation for 60 s (*t*_58_ = −2.61, *p* < 0.01) after the sleep disruption. Positive PANAS scores were significantly decreased (*t*_58_ = 227, *p* < 0.05) after the sleep disruption.

#### 3.1.2 Cohort Differences in Sleep Patterns After Sleep Disruption

RM‐ANOVA found a significant effect of time on the quality of sleep (*F*_3,165_ = 24.67, *p* < 0.001) and level of rest (*F*_3,165_ = 11.46, *p* < 0.001) reported in the sleep diaries, and post hoc tests (Bonferroni‐corrected) found that the quality of sleep (*p* < 0.001) and level of rest (*p* < 0.01) was significant during all three experimental nights compared to baseline. There were no significant differences in any of the sleep parameters measured by wrist actigraphy.

### 3.2 Sex‐Based Analyses

#### 3.2.1 Sex‐Based Difference in Baseline Measures

Males had significantly higher BMI compared to females [*t*(57) = 2.9, *p* < 0.01], with an average of 25.1 kg/m^2^ compared to 22.9 kg/m^2^ for females. Furthermore, males had significantly higher cPTT compared to females [*t*(57 = 2.7), *p* < 0.01] and higher CPM [*t*(57) = 2.2, *p* < 0.05] at baseline. There was a tendency for females to have higher PCS scores compared to males [*t*(57) = −2.0, *p* = 0.051]. There were no other sex‐specific differences in baseline measures, including baseline PSQI scores.

#### 3.2.2 Sex‐Based Differences in Outcomes After Sleep Disruption

**FIGURE 2:**
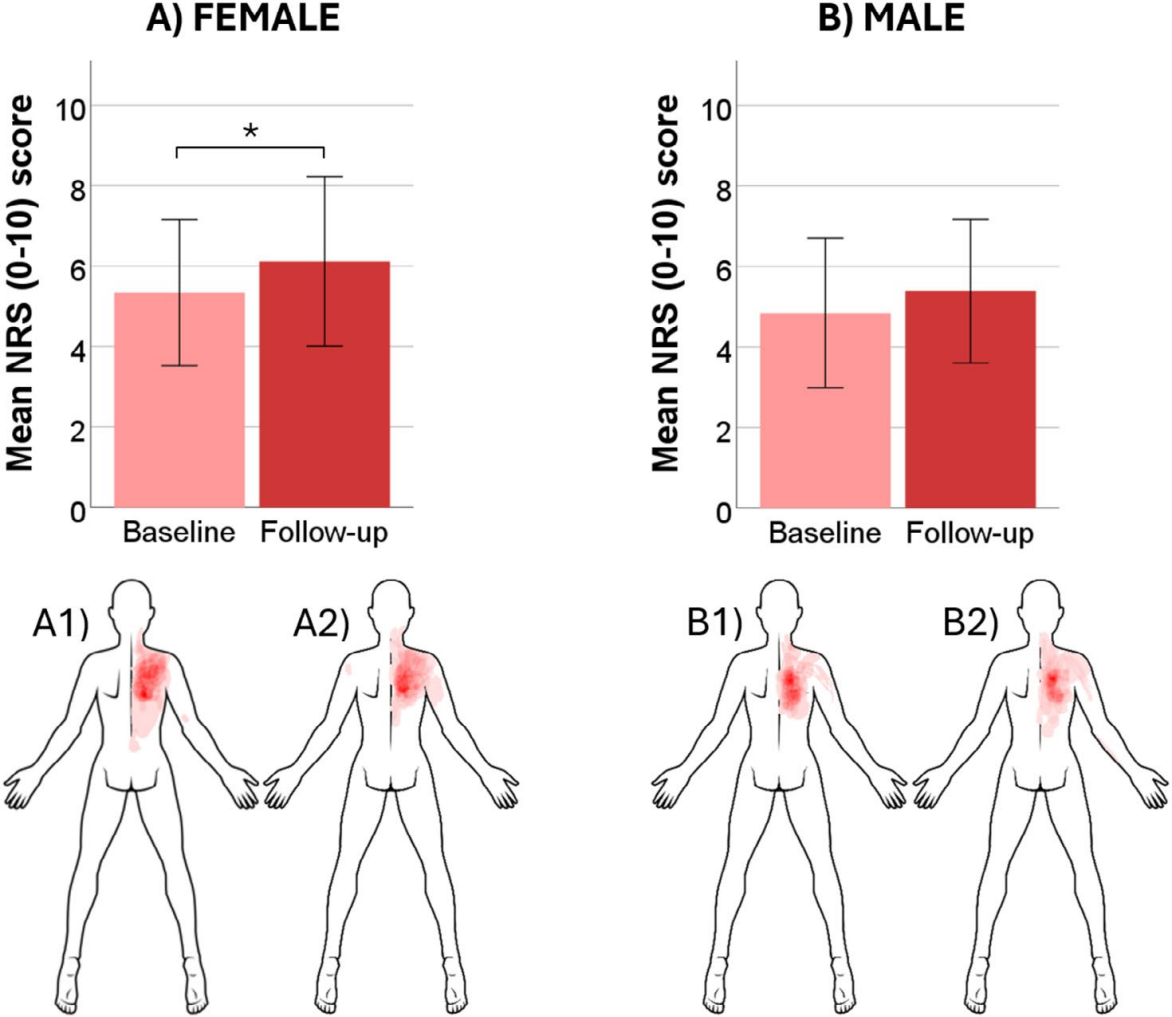
Overview of the sensitivity to 60‐s suprathreshold pressure stimulation for females (A) and males (B). The body charts illustrate an overlay of the distribution of the painful area marked by the participants for females at baseline (A1), females at follow‐up (A2), males at baseline (B1) and males at follow‐up (B2). Bar charts illustrate means ± 1 SD for the maximum pain rated during the stimulation at baseline and follow‐up for each group. NRS, numeric rating scale. *p < 0.05.

RM‐ANOVA found a significant effect of time on PTT (*F*_1,57_ = 5.6, *p* < 0.05), but no sex‐based differences were detected after Bonferroni correction. RM‐ANOVA found a significant effect of time on TSP (*F*_1,57_ = 4.6, *p* < 0.05) and post hoc tests (Bonferroni‐corrected) found that males had significantly increased TSP at follow‐up compared to baseline (mean difference = 0.46, *p* < 0.05). RM‐ANOVA found a significant effect of time on NRS rating during 120%PPT stimulation for 60 s (*F*_1,57_ = 6.7, *p* < 0.05) and post hoc tests (Bonferroni‐corrected) found that females had significantly increased NRS ratings at follow‐up compared to baseline (mean difference = 0.54, *p* < 0.05, Figure 2A). RM‐ANOVA found a significant effect of time on positive PANAS scores (*F*_1,57_ = 5.1, *p* < 0.05), but no sex‐based differences were detected after Bonferroni correction. Data from the QST measures and questionnaires for males and females can be seen in Table 1 and the pain distribution and intensities reported during 120%PPT can be seen in Figure 2 for females (Figure 2A) and males (Figure 2B).

**TABLE 1:** Summary of QST parameters [cuff pressure pain threshold (cPPT), cuff pain tolerance threshold (cPTT), temporal summation of pain (TSP), conditioned pain modulation (CPM), and 120% pressure pain threshold (PPT)] and Questionnaires (Positive and Negative Affective schedule (PANAS), Pain Catastrophizing Scale (PCS), and Pittsburgh Sleep Quality Index (PSQI)) data at baseline and follow‐up for females and males. Data is presented as the mean visual analogue scale (VAS), kilo pascal (kPa) pressure, numeric rating scale (NRS), marked area, or scores on questionnaires ± SD.

	Female, *N = 30*			Male, *N = 29*		
	Baseline	Follow‐up	Sig	Baseline	Follow‐up	Sig
CPPT, VAS (± SD)	32.5 ± 14.1	31.5 ± 13.4	—	35.0 ± 13.6	34.0 ± 13.2	—
CPTT, VAS (± SD)	76.3 ± 21.0	72.4 ± 23.4	—	89.9 ± 17.1	86.6 ± 20.0	—
TSP, VAS (± SD)	1.3 ± 1.0	1.4 ± 1.2	—	0.8 ± 1.3	**1.2 ± 1.2**	*
CPM, VAS (± SD)	7.2 ± 12.6	8.4 ± 13.7	—	16.1 ± 18.7	15.0 ± 14.7	—
120%PPT, NRS (± SD)	5.3 ± 1.8	**6.1 ± 2.1**	*	4.8 ± 1.9	5.4 ± 1.8	—
120%PPT AREA	0.6 ± 0.8	0.7 ± 0.6	—	0.4 ± 0.4	0.6 ± 0.5	—
Panas negative	17.1 ± 5.5	17.3 ± 5.5	—	16.8 ± 5.7	15.3 ± 4.6	—
Panas positive	29.4 ± 7.1	27.5 ± 6.2	—	30.6 ± 5.0	28.9 ± 5.5	—
PCS	10.4 ± 7.8	10.6 ± 7.1	—	6.7 ± 6.6	6.8 ± 6.1	—
PSQI	5.8 ± 3.1	6.0 ± 2.8	—	5.3 ± 2.3	5.9 ± 2.9	—

*Note:* * indicate significant differences (*p* < 0.05). Bold numbers indicate significant differences (*p* < 0.05) when compared to baseline (B).

#### 3.2.3 Sex‐Based Differences in Sleep Patterns After Sleep Disruption

RM‐ANOVA found a significant effect of time on the quality of sleep reported in the sleep diaries (*F*_3,162_ = 25.7, *p* < 0.001), and post hoc tests (Bonferroni‐corrected) found that males had significantly worse quality of sleep during all three experimental nights compared to baseline (*p* < 0.01), while females only had significantly worse quality of sleep during the first and second experimental nights (*p* < 0.001). Similarly, there was a significant effect of time on the level of rest reported in the sleep diaries (*F*_3,162_ = 11.9, *p* < 0.001), and post hoc tests (Bonferroni‐corrected) found that males were significantly less rested after the first and third experimental nights compared to baseline (*p* < 0.05), while females were significantly less rested after the first and second experimental nights compared to baseline (*p* < 0.01). RM‐ANOVA found a significant effect of time on the level of light sleep measured with wrist actigraphy (*F*_2,94_ = 3.8, *p* < 0.05), but no sex‐based differences were detected after Bonferroni correction. Data from self‐reported sleep quality, level of rest, and wrist actigraphy for males and females can be seen in Table 2.

**TABLE 2:** Summary of wrist actigraphy data and self‐reported sleep quality and level of rest (0–100) for males and females. Data is displayed for baseline (B) and each of the three experimental nights (N1, N2, and N3). N1, experimental night one; N2, experimental night two; N3, experimental night three. Data are presented as Mean Visual Analogue Scale (VAS), minutes (MIN), or numbers (N) ± SD.

	Female, *N = 30*				Male, *N* = 29			
	B	N1	N2	N3	B	N1	N2	N3
Sleep quality, VAS (± SD)	73.8 (± 14.3)	**57.9 (± 17.3)**	**60.5 (± 15.2)**	63.1 (± 14.9)	74.3 (± 19.0)	50.6 (± 19.4)	62.4 (± 15.9)	52.1 (± 20.2
Level of rest, VAS (± SD)	71.1 (± 17.5)	**57.1 (± 22.8)**	**58.2 (± 17.5)**	61.4 (± 15.4)	69.9 (± 20.1)	**59.0 (± 16.3)**	63.5 (± 16.8)	**51.2 (± 20.6)**
Light sleep, min (± SD)		258.8 (± 51.1)	274.2 (± 54.5)	255.6 (± 49.7)		264.2 (± 57.0)	281.2 (± 77.5)	237.3 (± 52.5)
Deep sleep, min (± SD)		76.4 (± 19.1)	82.9 (± 15.8)	79.7 (± 21.6)		74.8 (± 27.2)	72.9 (± 28.2)	73.1 (± 26.7)
REM sleep, min (± SD)		99.0 (± 28.6)	96.0 (± 31.0)	95.9 (± 32.1)		82.4 (± 42.6)	79.4 (± 26.9)	70.9 (± 28.6)
Awakenings, *N* (± SD)		28.2 (± 7.4)	33.1 (± 11.5)	28.8 (± 8.7)		30.7 (± 11.2)	32.5 (± 11.2)	31.7 (± 10.3)
Awakenings, min (± SD)		65.3 (± 15.1)	78.2 (± 22.6)	71.3 (± 27.2)		70.1 (± 22.3)	73.4 (± 24.8)	70.1 (± 18.6)
Total sleep, min (± SD)		424.2 (± 103.7)	451.4 (± 118.5)	430.8 (± 84.3)		421.4 (± 104.2)	433.4 (± 99.2)	381.3 (± 76.5)

*Note:* Bold numbers indicate significant differences (*p* < 0.05) when compared to baseline (B).

### 3.3 Sleep Quality‐Based Analyses

#### 3.3.1 PSQI‐Based Differences in Baseline Measures

Participants with poor sleep quality at baseline had lower baseline positive PANAS scores than participants with good sleep quality at baseline [*t*(57) = 2.3, *p* < 0.05]. There were no other PSQI‐based differences in baseline measures.

#### 3.3.2 PSQI‐Group Differences in Outcomes After Sleep Disruption

**FIGURE 3:**
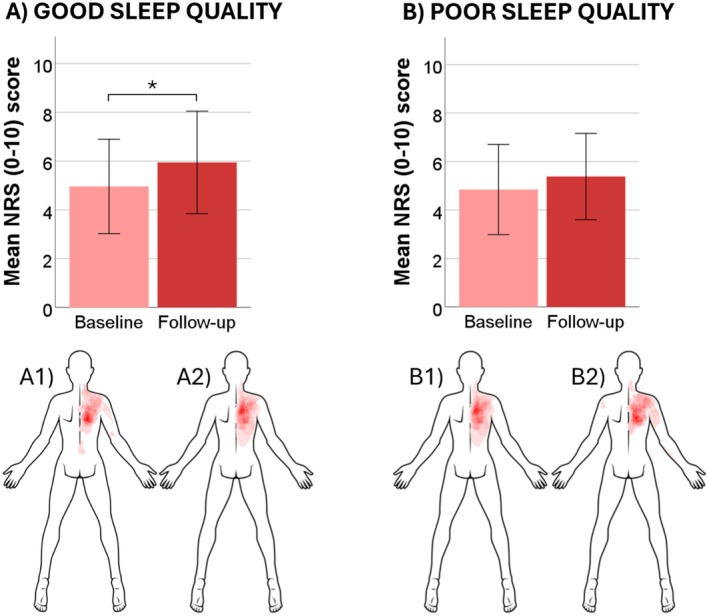
Overview of the sensitivity to 60‐s suprathreshold pressure stimulation for participants with good sleep quality (A) and poor sleep quality (B) based on baseline scores. The body charts illustrate an overlay of the distribution of the painful area marked by the participants for participants with good sleep quality at baseline (A1) and follow‐up (A2), and participants with poor quality of sleep at baseline (B1) and follow‐up (B2). Bar charts illustrate means ± 1 SD for the maximum pain rated during the stimulation at baseline and follow‐up for each group. NRS, numeric rating scale. *p < 0.05.

RM‐ANOVA found a significant effect of time on PTT (*F*_1,57_ = 5.1, *p* < 0.05) and TSP (*F*_1,57_ = 4.1, *p* < 0.05), but no PSQI‐based differences were detected after Bonferroni correction. RM‐ANOVA found a significant effect of time on NRS rating during 120%PPT stimulation for 60 s (*F*_1,57_ = 4.6, *p* < 0.05), and post hoc tests (Bonferroni‐corrected) found that participants with good baseline sleep quality had significantly higher NRS ratings at follow‐up compared to baseline (mean difference = 0.99, *p* < 0.01, Figure 3A). Data from the QST measures and questionnaires for the different sleep quality groups can be seen in Table 3 and the pain distribution and intensities reported during 120%PPT can be seen in Figure 2 for participants with good baseline sleep quality (Figure 3A) and poor baseline sleep quality (Figure 3B and Table 4).

**TABLE 3:** Summary of QST parameters [cuff pressure pain threshold (cPPT), cuff pain tolerance threshold (cPTT), temporal summation of pain (TSP), conditioned pain modulation (CPM), 120% pressure pain threshold (PPT) numeric ratings scale (NRS) score, and 120% pressure pain threshold (PPT) area] and Questionnaires [Positive and Negative Affective schedule (PANAS), Pain Catastrophizing Scale (PCS), and Pittsburgh Sleep Quality Index (PQSI)] data at baseline and follow‐up for the groups with good sleep quality (PSQI < 5) at baseline and poor sleep quality (PSQI ≥ 5) at baseline. Data are presented as mean Visual Analogue Scale (VAS), numeric rating scale (NRS), kilo pascal (kPa) pressure marked area, or scores on questionnaires ± SD.

	Good sleep quality, *N = 37*			Poor sleep quality, *N = 22*		
	Baseline	Follow‐up	Sig	Baseline	Follow‐up	Sig
CPPT, VAS (± SD)	35.9 ± 14.4	35.1 ± 14.6	—	30.1 ± 12.2	28.8 ± 9.6	—
CPTT, VAS (± SD)	85.6 ± 19.0	81.9 ± 23.7	—	78.7 ± 21.7	75.2 ± 20.8	—
TSP, VAS (± SD)	1.2 ± 1.1	1.5 ± 1.2	—	0.8 ± 1.3	1.0 ± 1.2	—
CPM, VAS (± SD)	11.6 ± 18.1	12.0 ± 15.2	—	11.5 ± 13.3	10.9 ± 13.4	—
120%PPT, NRS (± SD)	5.0 ± 1.9	**5.9 ± 2.1**	*	5.3 ± 1.7	5.4 ± 1.7	—
120%PPT area	0.5 ± 0.7	0.6 ± 0.5	—	0.6 ± 0.6	0.7 ± 0.8	—
Panas negative	16 ± 5.1	15.4 ± 4.9	—	18.5 ± 5.5	17.8 ± 5.4	—
Panas positive	31.4 ± 5.0	**28.2 ± 6.3**	*	27.6 ± 7.3	28.1 ± 5.0	—
PCS	7.6 ± 7.0	8.2 ± 6.4	—	10.2 ± 7.9	9.7 ± 7.7	—
PSQI	4.1 ± 1.0	**4.9 ± 2.2**	*	8.1 ± 2.7	7.6 ± 3.0	—

*Note:* * indicate significant differences (*p* < 0.05). Bold numbers indicate significant differences (*p* < 0.05) when compared to baseline (B).

**TABLE 4:** Summary of wrist actigraphy data and self‐reported sleep quality and level of rest (0–100) for the groups with good sleep quality at baseline (PSQI < 5) and poor sleep quality at baseline (PSQI ≥ 5). Data is displayed for each of the three experimental nights (N1, N2, and N3). N1, experimental night one; N2, experimental night two; N3, experimental night three. Data are presented as Mean Visual Analogue Scale (VAS), minutes (MIN), or numbers (*N*) ± SD.

	Good sleep quality, *N = 37*				Poor sleep quality, *N = 22*			
	B	N1	N2	N3	B	N1	N2	N3
Sleep quality, VAS (± SD)	75.1 (± 18.8)	**57.0 (± 18.7)**	**61.5 (± 17.7)**	**61.0 (± 18.4)**	72.2 (± 12.5)	**49.3 (± 17.3)**	**59.1 (± 11.9)**	**51.1 (± 16.6)**
Level of rest, VAS (± SD)	71.8 (± 19.8)	**61.6 (± 18.7)**	63.0 (± 17.6)	**59.2 (± 17.8)**	68.2 (± 16.9)	**51.1 (± 20.4)**	**55.5 (± 16.3)**	**49.4 (± 19.5)**
Light sleep, min (± SD)		268.6 (± 49.4)	280.5 (± 67.5)	235.0 (± 38.8)		257.7 (± 62.4)	268.8 (± 61.6)	266.2 (± 62.4)
Deep sleep, min (± SD)		80.4 (± 22.3)	80.3 (± 22.1)	73.8 (± 23.5)		70.7 (± 22.7)	73.1 (± 23.4)	79.3 (± 24.1)
REM sleep, min (± SD)		95.4 (± 35.4)	87.4 (± 30.3)	81.9 (± 29.4)		90.0 (± 40.3)	91.4 (± 29.4)	89.3 (± 36.5)
Awakenings, *N* (± SD)		29.1 (± 10.8)	28.5 (± 12.9)	26.7 (± 10.4)		29.2 (± 7.3)	34.5 (± 12.0)	32.2 (± 9.6)
Awakenings, min (± SD)		70.1 (± 18.2)	73.9 (± 26.0)	63.3 (± 20.4)		65.7 (± 22.2)	71.4 (± 23.4)	76.4 (± 27.8)
Total sleep, min (± SD)		434.6 (± 107.2)	446.0 (± 113.7)	389.7 (± 62.7)		418.4 (± 102.8)	433.2 (± 92.4)	434.8 (± 104.1)

*Note:* Bold numbers indicate significant differences (*p* < 0.05) when compared to baseline (B).

#### 3.3.3 PSQI‐Group Differences in Sleep Patterns After Sleep Disruption

RM‐ANOVA found a significant effect of time on the quality of sleep reported in the sleep diaries (*F*_3,162_ = 25.0, *p* < 0.001), and post hoc tests (Bonferroni‐corrected) found that participants with both good and poor baseline sleep quality had significantly worse quality of sleep during all three experimental nights compared to baseline (*p* < 0.01). Similarly, there was a significant effect of time on the level of rest reported in the sleep diaries (*F*_3,162_ = 12.3, *p* < 0.001) and post hoc tests (Bonferroni‐corrected) found that participants with good sleep quality at baseline were significantly less rested after the first and third experimental nights (0 < 0.05) while participants with poor sleep quality at baseline were significantly less rested after all three experimental nights (*p* < 0.05). There were no PSQI‐based differences in any sleep parameters measured by wrist actigraphy. Data from self‐reported sleep quality, level of rest, and wrist actigraphy for good and poor sleep quality can be seen in Table 2.

## 4 Discussion

This exploratory study found that three nights with experimental sleep disruption induced reduced pressure tolerance thresholds and increased sensitivity to suprathreshold stimulation, along with decreased positive affect scores. Further, sex and sleep quality‐specific changes in pain sensitivity and affective state were noted. TSP was significantly facilitated in males, and pain during suprathreshold stimulation was increased for females after the experimental sleep disruption. No differences in any QST parameters were found when comparing participants with good or poor sleep at baseline, but those with good baseline sleep rated the suprathreshold stimulation as more painful after the experimental sleep disruption. All participants demonstrated decreased sleep quality and level of rest after the sleep disruption, indicating successful implementation. There were both sex and PSQI‐group specific changes in self‐reported sleep quality and level of rest during the experimental nights.

### 4.1 Characteristics of Experimental Sleep Disruption

Previous literature has found a reciprocal correlation between sleep and pain (Koffel et al. 2016; Stocks et al. 2018), as well as psychological factors such as depression and anxiety (de Heer et al. 2014; Koffel et al. 2016). Given the prevalence of sleep disturbances in patients with chronic pain (Keilani et al. 2018; Tang 2008; Whale and Gooberman Hill 2022), the current study employed a sleep disruption protocol that mimicked the number of nightly awakenings reported by patients with chronic pain (Bjurstrom and Irwin 2016; Morin et al. 1998; Smith et al. 2000). This was chosen to investigate possible underlying mechanisms of poor sleep, which could exacerbate chronic pain. This will allow us to identify the unique contributions from sleep disruptions, without the many other complexities of chronic pain. The current study found decreased self‐reported sleep quality in all participants, which is in accordance with previous findings following experimental sleep disturbances (Hertel et al. 2023; Iacovides et al. 2017; Rosseland et al. 2018; Smith et al. 2019). In addition, the current study indicates that there is no different response in people with a different baseline quality of sleep, as both good groups reported a significant decrease in self‐reported quality of sleep and rest, but no differences were observed when assessing pre‐ and post‐PSQI scores. When investigating wrist actigraphy estimates of sleep architecture, those with good baseline sleep quality had significantly less light sleep on the final experimental night compared to the first. These findings indicate that the sleep disruption protocol applied in the current study impacts all participants independently of baseline sleep quality.

### 4.2 Sex‐Specific Changes After Experimental Sleep Disruption

Several studies have found an association between experimental sleep disturbance and increased pressure pain sensitivity in healthy individuals (Faraut et al. 2015; Schuh‐Hofer et al. 2013; Simpson et al. 2018; Staffe et al. 2019). In recent years, research has emerged showing the importance of sex in vulnerability to experimental sleep disturbances. Smith et al. (Smith et al. 2019) found increased secondary hyperalgesia in males and increased TSP in females after forced awakenings and sleep restriction and hypothesised that the experimental sleep disturbances were acting through distinct pathways in males and females (Smith et al. 2019). Further, a systematic review and meta‐analysis by Rouhi et al. (Rouhi et al. 2023) investigated the potential moderating effects of sex on sleep disturbance and pain perception. Sex was found as a moderator of changes in both CPM and TSP after experimental sleep disruption, with females having impaired CPM and increased TSP, while males tended to have a higher CPM and lower TSP (Rouhi et al. 2023). Similarly, a meta‐analysis by Babiloni et al. (2024) showed that studies dividing participants by sex found that experimental sleep disturbance led to impaired CPM exclusively in females. This contrasts with the findings of the current study, where the greatest impact of the experimental sleep disturbance was observed in males presenting with facilitation of TSP. In contrast, females showed increased pain ratings from suprathreshold painful pressure, and suprathreshold sensitivity is likely a combination of peripheral and central mechanisms (Doménech‐García et al. 2016; Laursen et al. 1999). Expansion of painful areas from suprathreshold pressure pain has been proposed to be a surrogate measure of convergence of adjacent receptive fields, and thus, a marker of dorsal horn windup. Nevertheless, while the distribution of the pain followed earlier reports of pain referral from the infraspinatus muscle (Arroyo‐Fernandez et al. 2020), no significant changes in size were detected in the current study for either sex. TSP is believed to be a human surrogate of wind‐up in the dorsal horn neurons (Latremoliere and Woolf 2009), which indicates that the current results reflect attenuated central pain mechanisms in males following the experimental sleep disruption, while females were more generally affected as indicated by increased NRS ratings from suprathreshold pressure pain. This could be explained by males having significantly lowered self‐reported sleep quality and level of rest on the day of follow‐up (after experimental night three) compared to females, which could indicate that the males in the current study were more sensitive to the experimental sleep disruption. Nevertheless, the differences observed in males in the current study are small and in discordance with existing evidence and should be interpreted as such. These findings, in combination with existing research, indicate that there is currently no clear evidence to support whether or not sleep disturbance protocols facilitate TSP and/or modulate other QST parameters, which is likely partly explained by the large heterogeneity in types of sleep disturbance and pain test modalities. Another potential issue could be individual variabilities in vulnerability to acute sleep loss, such as the sex‐related differences identified in the current study, and future studies should therefore attempt to identify factors that are important for why QST parameters could be altered after experimental sleep disruption.

### 4.3 Sleep Quality‐Specific Changes After Experimental Sleep Disruption

Previous studies employing various experimental sleep disturbances have shown significant heterogeneity (Chang et al. 2022; Herrero Babiloni et al. 2024). While this can likely be explained partly by the many types of sleep disturbances used and the different modalities used for pain sensitivity testing, participant characteristics should also be considered. Poor sleep quality (Hadi et al. 2019; Hodges et al. 2023), impaired CPM (McPhee et al. 2020; Ramaswamy and Wodehouse 2021; Zabala Mata et al. 2021), and facilitated TSP (McPhee et al. 2020) are all phenomenas often observed in patients with chronic pain. Further, experimental sleep disturbance protocols inducing short‐term poor sleep quality have been observed to induce changes in measures of central pain processing in healthy participants (Eichhorn et al. 2018; Simpson et al. 2018; Smith et al. 2019; Staffe et al. 2019), even though the effects are inconsistent across studies (Babiloni et al. 2020; Herrero Babiloni et al. 2024; Rouhi et al. 2023). The current study found decreased pressure pain tolerance following the experimental sleep disturbance and found an increase in sensitivity to suprathreshold pressure stimulation following the experimental sleep disturbance but only in participants with good baseline sleep quality, which could indicate that those with poor baseline sleep quality could be more resilient to experimental sleep disturbances or already affected. This is emphasised by the participants with good baseline sleep quality rated on the PSQI having significantly worse PSQI scores following the experimental sleep disruption.

Chronic pain and sleep disturbances often co‐occur (Sun et al. 2021) and have been linked to increased risk of concurrent anxiety and depression (Husak and Bair 2020). Several studies have identified changes in psychological parameters consequential to experimental sleep disturbances in healthy individuals, including increased symptoms of depression (Medic et al. 2017), symptoms of anxiety (Medic et al. 2017; Pires et al. 2016; Talbot et al. 2010), and lowered positive affect (Boon et al. 2023; Finan et al. 2016; Talbot et al. 2010). Huber et al. (Huber et al. 2022) proposed that the pronociceptive effects associated with poor sleep might be mechanistically explained by impairments in the emotional descending modulation of pain. The current study found lowered positive affect following the experimental sleep disruption in all participants. This could highlight how affective state is closely related to sleep quality, and preclinical studies have found the transitions in affective states after acute sleep deprivation to be mediated by dopaminergic brain circuits (Wu et al. 2024). In accordance with the findings of the current study, previous literature does not suggest a change in negative affect following sleep disruption (Boon et al. 2023; Finan et al. 2016; Lee et al. 2022; Talbot et al. 2010). Thus, research suggests that sleep is crucial for cognitive functions, including emotion regulation (Palmer and Alfano 2017), emphasising the importance of good sleep hygiene for overall cognitive, emotional, and physical well‐being.

### 4.4 Limitations

The current study used the Fitbit Charge 4 to measure sleep/awake states, awakenings, and sleep stages in the participant's own home. The reliability of the device is not tested for at‐home use and lacks specificity in the assessment of the specific stages of sleep (Schyvens et al. 2024), which could obscure any changes in sleep architecture from the sleep disruption in the current study. Furthermore, no baseline Fitbit data was collected before the sleep protocol, which hinders any conclusions on changes in sleep architecture during the intervention compared to regular sleep. Nevertheless, baseline sleep characteristics were reported through self‐rated questionnaires in the form of PSQI and follow‐up questions regarding sleep quality and level of rest.

The sample in the current study consists of young adults, as this group seems to be particularly vulnerable to the effects of poor sleep, partly due to an increased need for sleep compared to older adults (Hirshkowitz et al. 2015) but also interference with developmental processes (Benitez and Gunstad 2012; Kopasz et al. 2010). Nevertheless, this limits the generalizability of the results to the wider population, and the effects of experimental sleep disruption should be investigated in broader samples in future research. Further, the current sample consisted of healthy pain‐free individuals, which might not compare to chronic pain patients, who might have trouble reinitiating sleep after an awakening, and thus cause increased sleep deprivation from the awakenings. Therefore, future studies should attempt to better mimic the complex presentation of the sleep disturbances experienced by individuals with chronic pain, which could be achieved by adding a painful condition, such as delayed‐onset muscle soreness, to the model. The current study is exploratory, as a prior sample size calculation was not completed. This is important, as the current analysis might be underpowered, and this should be considered when interpreting the results.

## 5 Conclusions

The exploratory current study successfully employed experimental sleep disruption in 59 healthy participants, characterised by lowered sleep quality and level of rest. Males were found to have an increased TSP and females to have an increased pain response to suprathreshold stimuli, suggesting that males and females respond differently to a three‐night sleep disruption protocol. Further, participants with good sleep quality at baseline had an increased response to suprathreshold stimulation, suggesting that the sleep disruption predominantly affects the pain sensitivity of those who usually sleep well. Finally, having good or poor sleep quality at baseline both demonstrated significant decreases in sleep quality after the sleep disruption, suggesting that baseline sleep quality does not modulate individual susceptibility to sleep deterioration during experimental sleep disruption.

## Author Contributions

This study was designed by Kristian Kjær‐Staal Petersen, Emma Hertel, Rocco Giordano, Elisabet Dortea Ragnvaldsdóttir Joensen, Laura Frederiksen, Signe Vindbæk Frederiksen, and Emilie Stjernholm Valeur. The experiments were performed by Elisabet Dortea Ragnvaldsdóttir Joensen, Laura Frederiksen, Signe Vindbæk Frederiksen, Emilie Stjernholm Valeur, and Emma Hertel. The data were analysed by Emma Hertel and Kristian Kjær‐Staal Petersen, and the results were critically examined by all authors. Elisabet Dortea Ragnvaldsdóttir Joensen, Laura Frederiksen, Signe Vindbæk Frederiksen, and Emilie Stjernholm Valeur prepared the initial draft of the manuscript, which was edited by Kristian Kjær‐Staal Petersen, Emma Hertel, Elisabet Dortea Ragnvaldsdóttir Joensen, Laura Frederiksen, Signe Vindbæk Frederiksen, and Emilie Stjernholm Valeur. All authors have approved the final version of the manuscript and agree to be accountable for all aspects of the work.
